# Comprehensive selection of reference genes for quantitative RT-PCR analysis of murine extramedullary hematopoiesis during development

**DOI:** 10.1371/journal.pone.0181881

**Published:** 2017-07-21

**Authors:** Giuliana Medrano, Peihong Guan, Amanda J. Barlow-Anacker, Ankush Gosain

**Affiliations:** 1 Division of Pediatric Surgery, Department of Surgery, University of Tennessee Health Sciences Center, Memphis, Tennessee, United States of America; 2 Department of Surgery, University of Wisconsin School of Medicine and Public Health, Madison, Wisconsin, United States of America; 3 Children’s Foundation Research Institute, Le Bonheur Children’s Hospital, Memphis, Tennessee, United States of America; Northwestern University, UNITED STATES

## Abstract

The purpose of this study was to perform a comprehensive evaluation and selection of reference genes for the study of extramedullary hematopoiesis during development and the early post-natal period. A total of six candidate reference genes (*ACTB*, *GAPDH*, *HPRT1*, *PPID*, *TBP*, *TUBB3*) in four organs (heart, liver, spleen, and thymus) over five perinatal time points (Embryonic days 14.5, 16.5, 18.5, Post-natal days 0, 21) were evaluated by quantitative real-time PCR. The expression stability of the candidate reference genes were analyzed using geNorm, NormFinder, Bestkeeper, Delta CT method, and RefFinder software packages. Detailed methodology for isolation of high quality/purity RNA and analysis is presented. Detailed analysis demonstrated that *TBP* is the best single reference gene for embryonic samples and *HPRT1* is the best single reference gene for post-natal and pooled embryonic and post-natal samples. Organ-level analysis demonstrated that *HPRT1* was the most suitable reference gene for heart, liver and thymus samples, while *TBP* was the best candidate for spleen samples. In general, *TUBB3* was consistently the least stable gene for normalization. This is the first study to describe a systematic comprehensive selection of reference genes for murine extramedullary hematopoietic tissues over a developmental time course. We provide suggested reference genes for individual tissues and developmental stages and propose that a combination of reference genes affords flexibility in experimental design and analysis.

## Introduction

Extramedullary hematopoiesis (EH), the process of blood cell differentiation from hematopoietic stem cells occurring in organs outside of the bone marrow (BM), is a normal component of pre-natal development. However, postnatally and through adulthood, physiologic hematopoiesis takes place primarily in the BM while EH is considered pathologic. Post-natal EH typically occurs in the setting of insufficient BM function, ineffective hematopoiesis, hematologic disorders, stromal disorders, or systemic inflammatory conditions [1]. Despite the common nature of these pathologic conditions, there is a paucity of literature addressing techniques for studying both physiologic and pathologic EH.

In humans, hematopoiesis starts at 2 weeks’ gestation in the paraaortic splanchnopleura of the yolk sac and later in the aorta-gonad-mesonephros [2–5]. Hematopoiesis then appears in the liver at approximately 5 weeks gestation [6] and continues with the liver as the primary site of hematopoiesis until 4.5 months of gestation, at which point BM hematopoiesis increases and exceeds that of the liver [7, 8]. Hematopoiesis also occurs in the pre-natal spleen until the second trimester. The thymus is also an hematopoietic organ, maturing by 15 weeks of gestation [9]. The thymus primarily produces T lymphocytes; however, other hematopoietic cell types can also be found in this tissue [10–12]. The different developmental stages of the embryo and adult present variations in the cell number, total and poly (A) RNA contents [13–15]. Understanding the biological dynamics of EH during early development may yield insights into the complex molecular pathways controlling hematopoietic development through the adult life.

EH has been observed in a number of hematological disorders including myelofibrosis, lymphoma, leukemia, and chronic hemolytic anemias such as sickle cell anemia, and thalassemia [16, 17]. Induced EH also appears during immune responses to a wide variety of microbiological challenges (e.g *Toxoplama gondii* [18], ehrlichiosis [19] schistosomiasis [20], cytomegalovirus [21]). These inflammatory responses are associated with profound changes in BM that impact hematopoietic differentiation and mobilization leading to the induction of EH. While programmed EH is required to replace the lack of hematopoietic activity in BM, excessive disease-associated EH may result from chronic inflammation [17]. Disease-associated EH has been observed in diverse organs including liver, spleen, lymph nodes, and heart [1].

Quantitative Real-Time Polymerase Chain Reaction (qPCR) is the optimal method for rapid and reliable quantification of mRNA transcription. Relative quantification is performed by comparing expression levels of the target gene of interest to internal reference genes. For studies requiring precise comparisons of mRNA transcription in different samples or tissues it is crucial to choose the appropriate reference gene(s) [22]. The use of an inappropriate reference gene may lead to erroneous normalization and flawed conclusions [23]. Since there is no universal reference gene with a constant expression in all tissues, reference genes need to be selected and validated for each specific application [24]. Technical factors, such as RNA quality, cDNA synthesis, primer design, and the PCR protocol can dramatically influence qPCR results. The primary goal of the current study was to compare the expression of a group of commonly used housekeeping genes across a series of embryonic and post-natal developmental stages in sites of EH in order to identify the most stable gene(s) for normalization.

## Material and methods

### Animals and tissue collection

This study was carried out in strict accordance with the recommendations in the Guide for the Care and Use of Laboratory Animals of the National Institutes of Health. The protocol was approved by the Animal Care and Use Committees of the University of Wisconsin-Madison (Protocol #M01394) and University of Tennessee Health Science Center (Protocol #16–021). C57BL/6J wild type (WT) mice were obtained from Jackson Laboratories (Stock Number: 000664) and a breeding colony established.

Animals were time mated and the day the vaginal plug was identified defined as embryonic day (E) 0.5. Embryos were isolated from pregnant dams that had been anesthetized with isoflurane and humanely euthanized by cervical dislocation. Postnatal (P) animals were humanely euthanized using isoflurane and cervical dislocation. Tissues (liver, spleen, thymus, heart) were collected, snap frozen in liquid nitrogen and stored at -80°C. Three biological replicates of each tissue type were collected at five developmental stages: E14.5, E16.5, E18.5, P0, and P21.

### RNA isolation, quantification, purity and integrity

Total RNA was isolated from frozen tissue using silica-membrane based kits from Qiagen. RNeasy Micro kit (Qiagen, Hilden, Germany) was used for all embryonic ages and P0, except for liver samples for which we used the RNeasy Mini kit in all ages. P21 samples were isolated using RNeasy Mini kit (Qiagen, Hilden, Germany). Beta-mercaptoethanol (BME) was added to the lysis buffer to prevent degradation by RNases. Fully automated RNA isolation (QIAcube, Qiagen, Hilden, Germany) was used for samples at E18.5, P0, and P21. E14.5 and E16.5 samples were processed manually. On-column DNAse digestion was also performed for all samples. RNA concentration and purity (260/280nm ratio) was determined using Nanodrop 2000 spectrophotometer (Thermo Fisher Scientific, Wilmington, DE). RNA integrity was determined by capillary electrophoresis (Bioanalyzer 2100, Agilent Technologies, Waldbronn, Germany).

### cDNA synthesis and pre-amplification

The cDNA synthesis was carried out in the presence of ProtoScript II First Strand cDNA synthesis kit using 150 ng RNA, 1 μl of oligo d(T)23 VN (50 μM) and 1 μl of random primer mix (60 μM) in a final volume of 20 μl according manufacturer’s instructions (New England BioLabs, Ipswich, MA). To pre-amplify small amounts of cDNA we used Taqman PreAmp Master Mix (Applied Biosystems, Foster City, CA). The PreAmp mix was performed using a primer pool consisting of 50 nM final concentration of each primer, cDNA at a final concentration of 22.5 ng in a final volume of 10 μl. The pre-amplification reaction was conducted as follows: 1 cycle of 95°C for 10 minutes and 14 cycles of 95°C for 15 seconds and 60°C for 4 minutes. After pre-amplification samples were stored at -20°C.

### Candidate reference genes

Six reference genes with distinct functions in live cells were selected for evaluation of their expression profile (Table 1): Beta-actin (*ACTB*), Glyceraldehyde 3-phosphate dehydrogenase (*GAPDH*), Hypoxanthine-guanine phosphoribosyltransferase (*HPRT1*), Peptidylprolyl isomerase D (*PPID*), TATA box binding protein (*TBP*), and Tubulin beta 5 (*TUBB3*). The NCBI reference number of the gene used to design the primers, primer sequence, Universal ProbeLibrary (UPL) (Roche) probe number are detailed in Table 1. The amplicon size was kept in a narrow size range (61–123 bp) to ensure similar PCR efficiencies for comparison between assays. The UPL probes were labeled at the 5’ end with fluorescein (FAM) and at the 3’ end with a dark quencher dye. Locked Nucleic Acids (LNA) incorporated into the sequence of each UPL probe to increase specificity.

**Table 1:** Summary of reference genes selected for analysis.

Gene symbol	Name	Gene function	NCBI Reference #	Primers Left/Right	UPL Probe #	Amplicon (nt)
*ACTB*	Beta-actin	Cytoskeletal structural protein	NM_007393.1	aaggccaaccgtgaaaagat/gtggtacgaccagaggcatac	56	110
*GAPDH*	Glyceraldehyde-3-phosphate dehydrogenase	Oxidoreductase in glycolysis and gluconeogenesis	MGI: 95640/ENSMUSG00000057666	tgtccgtcgtggatctgac/cctgcttcaccaccttcttg	80	75
*HPRT1*	Hypoxanthine guanine phosphoribosyl transferase 1	Purine synthesis in salvage pathway	NM_013556.2	tcctcctcagaccgctttt/cctggttcatcatcgctaatc	95	90
*PPID*	Peptidylprolyl isomerase D	Modulatory component of the mitochondrial permeability transition pore	NM_026352.2	atggtgaaaaacctgccaaa/catcctcagggaagtctgga	21	123
*TBP*	TATA box binding protein	RNA polymerase II, transcription factor	ENSMUST00000014911.3	ggtcgcgtcattttctcc/gggttatcttcacacaccatga	107	61
*TUBB3*	Tubulin, beta 5	Structural component of microtubules	NM_023279.2	gcgcatcagcgtatactacaa/ttccaagtccaccagaatgg	104	73

### Primer/Probe design and real-time PCR

We used ProbeFinder (Universal ProbeLibrary Assay Design Center, Roche, Indianapolis, IN) for mouse species to design primers and UPL probes for six reference genes (Table 1). Real-time PCR reactions were prepared in a final volume of 10 μl containing 0.1 μl of UPL probe (10 μM), 0.2 μl of left (10 μM) and right (10 μM) primers, 5ul of 2X Kapa probe fast qPCR master mix (Kapa Biosystems, Wilmington, MA), 2 μl of cDNA and 2.7 μl molecular grade water. Three biological replicates for each sample were run in a 384-well white plate optimized for Roche 480 Light Cycler (Phenix Research Products, Candler, NC). Non-template controls were run in triplicate. Interplate calibrators (IPC) were also included in triplicates in each plate as a method for inter-run variation compensation (Tataa Biocenter AB, Goteborg, Sweden). All plates were run in Roche LightCycler 480 machine using the Mono Color Hydrolysis Probe/UPL program: 1 cycle of pre-incubation step at 95°C for 5 minutes, 45 cycles of denaturation step at 95°C for 10 seconds, 60°C for 30 seconds, and 70°C for 10 seconds) with acquisition mode “single” at the last step of amplification.

### qPCR efficiency

RNA isolation and cDNA synthesis were processed independently for each sample. Three independent cDNAs were prepared for each sample. cDNA from each organ was pooled and three pools of cDNA were obtained for determining qPCR efficiency. All the pooled specimens were tested using five serial dilutions: 50, 5, 0.5, 0.05, and 0.005 ng (final concentration of cDNA). Efficiency of amplification for each gene was determined by analysis of the slope of the standard curve (Light Cycler 480 SW 1.5.1, Roche Applied Science, Penzberg, Germany). The highest possible quality PCR reaction produces a standard curve with an efficiency of 2, which refers to the number of target nuclei acid doubles with each amplification cycle. The error is the mean squared error of the single data points fit to the regression line. An error < 0.2 was considered acceptable.

### Expression stability analysis and software programs

To analyze the gene expression stability of the candidate reference genes, we utilized geNorm [25] NormFinder [26], Bestkeeper [27], Delta CT method [28], and RefFinder [29]. GenEx Pro ver 6.1.0.757 software (MultiD Analyses AB, Goteborg, Sweden) was used to calculate the Accumulated Standard Deviation (Acc. SD) and to verify the geNorm and Normfinder results.

In the case of geNorm analysis, stability of reference genes is determined by calculating the M value (average pairwise variation analysis). The most stable gene yields the lowest M value and the two most stable reference genes are determined by stepwise exclusion of the least stable gene. In NormFinder software, the stability value of the candidate genes is calculated according to their intra- and intergroup variations. Stably expressed genes will have low stability values. BestKeeper utilizes a two-way ranking: Pearson’s correlation coefficient and BestKeeper computed SD values. Gene ranking by Delta Ct method is calculated by pair-wise comparisons. Using raw Ct values, the average SD of each gene is inversely proportional to gene stability. The more stable gene has the lowest CV and SD. RefFinder integrates all four programs (geNorm, NormFinder, BestKeeper, and Delta Ct method) and calculates a weighted geometric mean for a ranking of the best reference genes. Means of different groups were compared and analyzed using the Student’s t-test. Standard error (SEM) was used. GraphPad Prism version 7.0b (GraphPad Software) was used for the box plot, statistical analysis and graphs plotting.

## Results

### Quality of RNA samples

**Fig 1:**
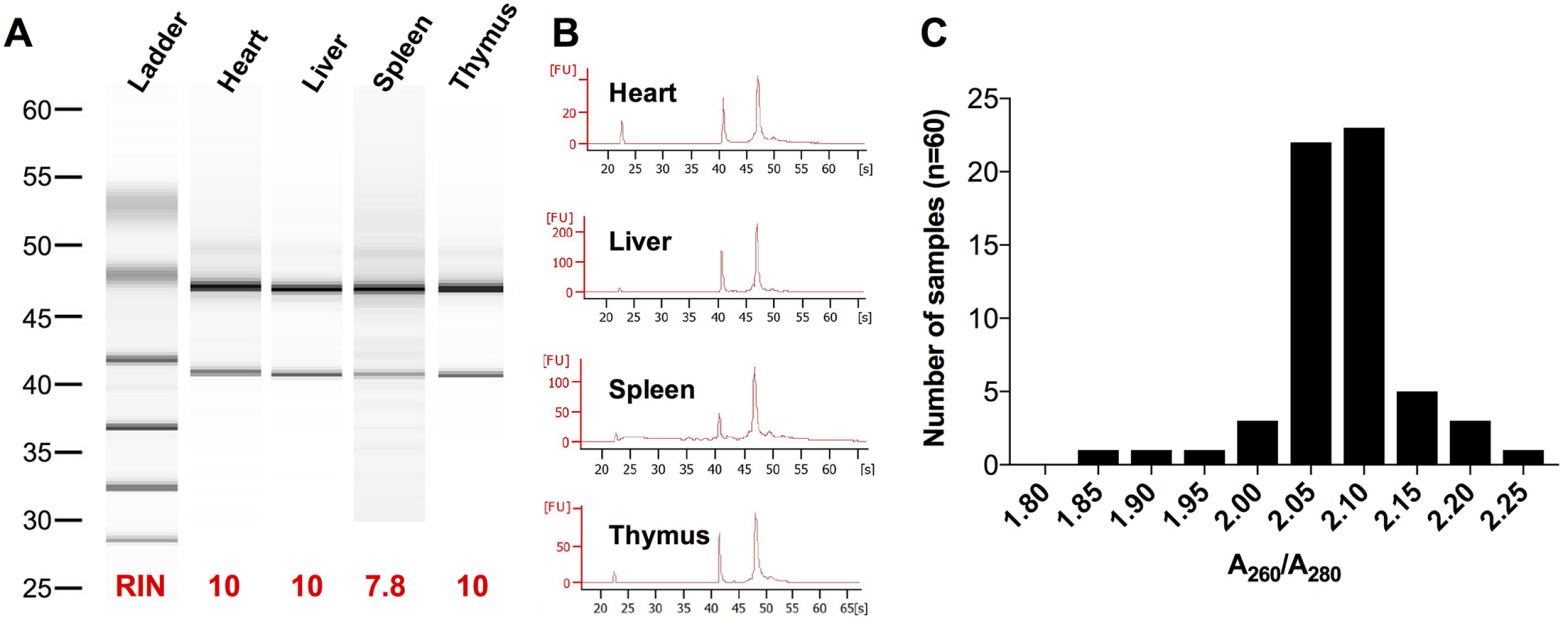
RNA quality and purity. RNA Integrity Number (RIN) values obtained from Agilent 2100 Bioanalyzer System was utilized to test the quality of the RNA samples and the Histogram of Frequency Distribution of the A260/A280 ratio was used to indicate the purity of the samples. **(A)** Electrophoresis of representative sample types allows visual inspection of RNA quality of the different tissues (heart, liver, spleen, and thymus). **(B)** The electropherogram shows 18S (left) and 28S (right) peaks indicative of RNA integrity of the different organs. **(C)** Histogram of frequency showing the distribution of the purity of RNA was performed using the ratio of absorbance at A260 nm/absorbance at A280 nm. X axis shows the A260/A280 for the 60 samples tested independently.

Isolation of quality of RNA including integrity and purity is crucial for comparative gene expression experiments. We used a micro-capillary electrophoretic RNA separation to estimate the RNA integrity number (RIN) and the ratio of 28S:18S ribosomal RNA by Agilent 2100 Bioanalyzer [30]. RIN values range from 7.8 (spleen) to 10 (heart, liver and thymus) (Fig 1A). Because splenic samples have high nuclease and nucleic acid content, BME was added to the lysis buffer during RNA isolation to minimize RNA degradation. Splenic sample RIN without BME ranged from 2.6–3.7. Representative electropherograms showing the intensity of the two ribosomal peaks, indicative of good RNA integrity, for all four organ types are shown in Fig 1B. RNA purity can be measured by the ratio of A260 nm/A280 nm absorbance, with a value of 2 +/- 0.3 indicating pure RNA. The distribution of A260/280 values for all 60 samples (heart, liver, spleen and thymus of ages E14.5, 16.5, 18.5, P0, and P21) is shown in Fig 1C.

### PCR performance and efficiency

Experiments involving embryonic organs often rely on small amounts of source tissue. To overcome this limitation, we utilized a pre-amplification kit (PreAmp, Applied Biosystems) to amplify small amounts of cDNA into many real-time PCR reactions, thereby allowing testing of all selected reference genes. An initial test was done in triplicates using P0 samples (heart, liver, spleen, and thymus) and *HPRT1* reference. We compared ten serial dilutions (original cDNA, PreAmp pure cDNA, and PreAmp cDNA dilutions 1:5, 1:20, 1:50, 1:100, 1:200 1:400, 1:800, and 1:1,600) to confirm that there is no variation in the expression results introduced by the pre-amplification step and to determine which is the best dilution to use for further experiments (data not shown). We selected the PreAmp 1:100 cDNA dilution for further analysis.

Using a standard curve generated by serial dilutions of cDNA from a pool of embryonic tissue and a pool of post-natal tissue, efficiency was calculated for each of the 6 candidate genes. PCR efficiency ranged from 1.9 to 2.2 (Table 2), indicating good to excellent efficiency for all 6 primers.

**Table 2:** PCR efficiency of six reference genes.

Gene symbol	Type of Sample	Efficiency	Standard Error	Slope*
*ACTB*	Post-natal	2.002	0.01820	-3.317
	Embryo	2.014	0.00417	-3.288
*GAPDH*	Post-natal	2.175	0.07490	-2.964
	Embryo	1.862	0.04160	-3.704
*HPRT1*	Post-natal	2.044	0.06570	-3.220
	Embryo	2.024	0.01620	-3.265
*PPID*	Post-natal	1.972	0.01030	-3.390
	Embryo	1.997	0.00594	-3.328
*TBP*	Post-natal	1.970	0.00962	-3.396
	Embryo	2.042	0.01750	-3.225
*TUBB3*	Post-natal	2.052	0.17700	-3.203
	Embryo	1.979	0.00903	-3.374

*Slope calculated between log_10_RNA (ng/reaction) and Cp values. Values were calculated using LigthCycler480 (Roche).

### Reference gene expression stability over time

**Fig 2:**
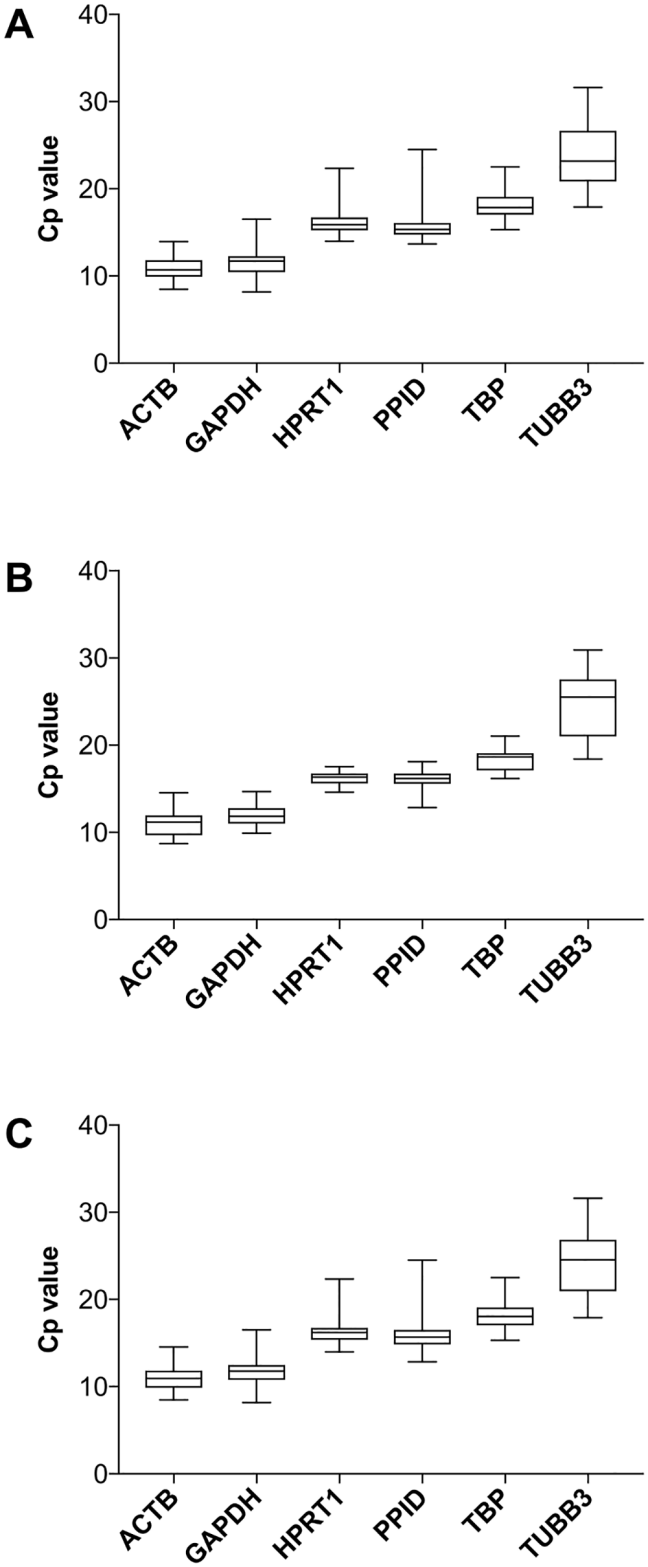
Reference gene expression stability over time. Distribution of the raw Cp values for embryonic organs (A), post-natal (B) and pooled (C). The box plot of the real-time PCR compiles 60 raw Cp values for each gene. All three plots demonstrate a similar trend. A highest variation is showed in *TUBB3* compared with the other genes.

In order to identify the reference gene(s) with the lowest variation in expression among different tissues and different developmental time points, raw Cp values of the 6 reference genes were plotted for embryonic samples (Fig 2A), post-natal samples (Fig 2B) and pooled embryonic and post-natal samples (Fig 2C). All three plots demonstrate a similar trend, with the highest variation in *TUBB3* compared with the other genes.

In order to detail the temporal variability in our selection of reference genes, we analyzed the genes based on the ages E14.5, E16.5, E18.5, P0, and P21 using pooled cDNA from the four organs of interest (heart, liver, spleen, thymus) at those time points (S1 Fig). We found that expression of each individual reference gene is near-constant across the developmental stages, validating the grouping of time points into embryonic, post-natal, and pooled. Additionally, *ACTB*, *GAPDH*, *HPRT1*, and *TBP* demonstrate very similar levels of expression to one another over time. However, *TUBB3* and *PPID* differ from the other candidate reference genes, demonstrating consistently higher gene expression over the time course.

**Fig 3:**
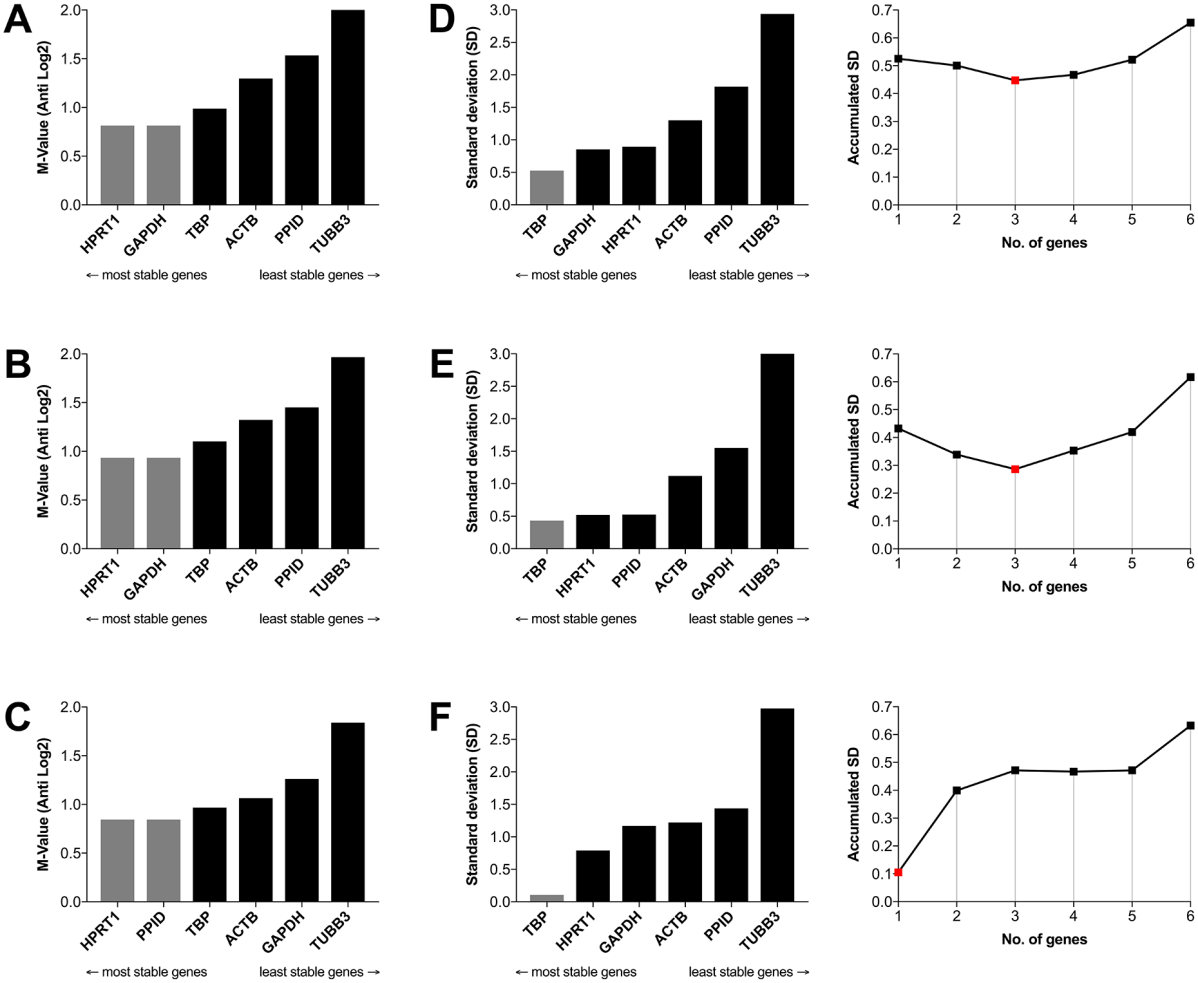
Stability analysis of the six reference genes. geNorm (A-C) and NormFinder (D-F) were used to identify reference genes for normalization. The 1^st^ row are embryonic organs (A and D), the 2^nd^ row shows post-natal organs (B and E), and 3^rd^ row represent pooled organs (C and F). The accumulated SD calculated in NormFinder is shown in the right column.

In order to determine the appropriate number of reference genes to utilize for accurate normalization across tissues and time points, we used the geNorm and NormFinder modules of GenEx (Fig 3). geNorm ranks reference genes based on their expression stability (M), where a low value is the more stable than a high value (Fig 3A–3C). The algorithm uses paired expression values to sequentially eliminate the gene that shows the highest variation relative to the other genes, resulting in a pair of recommended reference genes. As shown in the Fig 3, the order of gene stability ranking is similar for embryonic and post-natal samples. *HPRT1* and *GAPDH* (M = 0.81 for embryonic; M = 0.84 for post-natal) were the most stable pair of reference genes (Fig 3A and 3B). However, in the case of pooled samples the most stable pair of genes were *HPRT1* and *PPID* (M = 0.93, Fig 3C). *TUBB3* was the reference gene with the least stable expression for embryonic (M = 2.02), post-natal (M = 1.84), and pooled (M = 1.97).

NormFinder was used to calculate the expression stability value (standard deviation) for the six candidate reference genes. NormFinder identified *TBP* as the single best candidate gene for embryonic (SD = 0.52), post-natal (0.43), and pooled specimens (0.10) (Fig 3D–3F). NormFinder also calculates the Accumulated Standard Deviation (Acc SD) in order to determine the optimal number of reference genes (Fig 3). When we analyzed embryonic or post-natal samples, the Acc SD indicated the lowest values for a combination of three reference genes for embryonic (*TBP*, *GAPDH*, *HPRT1*, Acc SD = 0.4477) and post-natal (*TBP*, *HPRT1*, *PPID*, Acc SD = 0.2862) samples, but only a single gene (*TBP*, Acc SD = 0.1050) for the pooled specimens.

A comprehensive ranking of the six candidate reference genes for embryonic, post-natal and pooled samples were calculated using geNorm, NormFinder, BestKeeper, Delta Ct, and RefFinder (Table 3). The results indicate that *HPRT1* and/or *TBP* were the most frequently identified single best gene for embryonic samples. *HPRT1* and/or *PPID* were the most frequent single best gene identified in post-natal samples. *HPRT1* and/or *TBP* were the most frequently identified best reference genes for pooled samples. RefFinder analysis involves an integrated calculation including geNorm, NormFinder, BestKeeper and Delta Ct methods. This analysis demonstrated that *TBP* is the best reference gene for embryonic samples and *HPRT1* is the best reference gene for post-natal and pooled samples.

**Table 3:** Comprehensive ranking of candidate reference genes for embryonic, post-natal and pooled samples.

	geNorm	NormFinder^a^	BestKeeper^b^	Delta CT method	RefFinder	Best pair^c^	Best trio^c^
**Embryonic**	*HPRT1/GAPDH*	*TBP*	*ACTB*	*TBP*	*TBP*	*HPRT1*/*TBP*	*HPRT1*/*TBP*/*GAPDH*
**Post-natal**	*HPRT1/PPID*	*TBP*	*HPRT1*	*HPRT1*	*HPRT1*	*HPRT1*/*PPID*	*HPRT1*/*PPID*/*TBP*
**Pooled**	*HPRT1/GAPDH*	*TBP*	*HPRT1*	*TBP*	*HPRT1*	*HPRT1*/*TBP*	*HPRT1*/*TBP*/*GAPDH*

^a^ Best combination of two genes determined considering the intragroup and intergroup variation.

^b^ Best gene considering the correlation values (r).

^c^ Best combination is based in a visual inspection of all the ranks generated by the four methods.

### Gene expression analysis by organ

**Fig 4:**
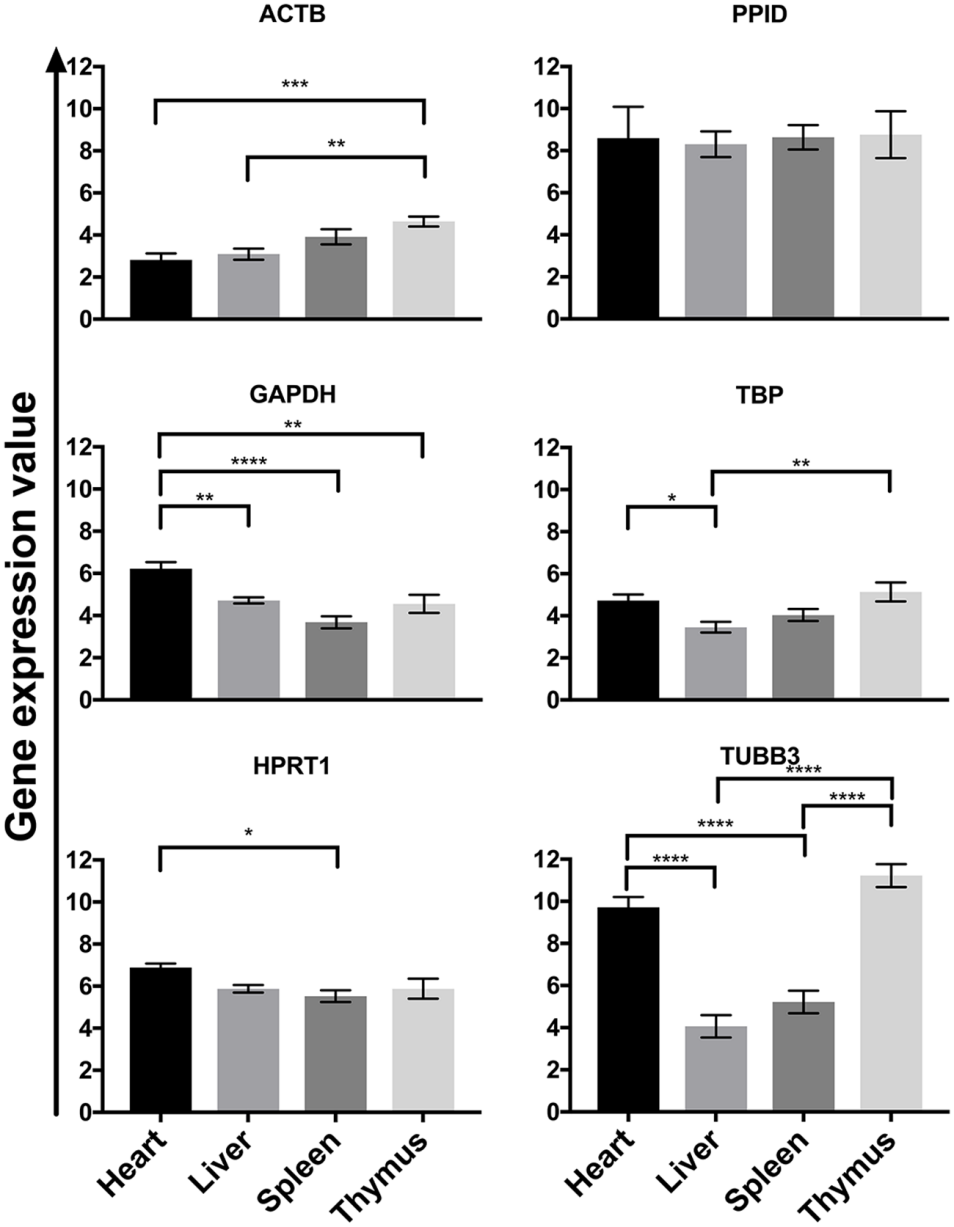
Gene expression analysis by organ. Expression profiles of individual reference genes in each of the tissues (heart, liver, spleen, thymus) using pooled samples representing all time points. Fig shows the mean +/- SEM of 15 samples for each tissue type. * *p* ≤ 0.05, ** *p* ≤ 0.01, *** *p* ≤ 0.001, **** *p* ≤ 0.0001.

In order to understand tissue level variability in our selection of reference genes, we analyzed the genes based on the four organs of interest (heart, liver, spleen, thymus) (Fig 4). By using a pool of cDNA from each of the developmental stages for each organ, we found significant differences in expression within organs in all of the reference genes except *PPID*, for which there were no significant differences between organs. *TUBB3* demonstrated high degrees of variation between organs.

The results of NormFinder, geNorm, DeltaCT, and RefFinder demonstrate consensus, indicating that *HPRT1* is the most stable reference gene in liver and thymus (Table 4). However, BestKeeper indicates *HPRT1* was the second most stable gene for liver and thymus. In heart, *HPRT1* is the most stable reference gene according geNorm, BestKeeper, and RefFinder. While NormFinder results show *ACTB* as the most stable gene in heart, Delta CT methodology demonstrates that *TBP* is the most stable gene. The spleen has either *GAPDH* (NormFinder, geNorm) or *TBP* (geNorm, BestKeeper, Delta CT, and RefFinder) as the best reference gene. *TUBB3* was consistently the least stable or the second least stable in all organs. By using RefFinder we were able to identify consensus reference genes between the 4 different algorithms. Based on these analyses, *HPRT1* was the most suitable reference gene for heart, liver and thymus samples, while *TBP* was the best candidate for spleen samples (Table 4).

**Table 4:** Ranking of the candidate reference genes by organ type.

NormFinder		geNorm		BestKeeper		Delta CT		RefFinder	
Stability value	Ranking	M value	Ranking	STDEV (+/- Cp)	Ranking	Average of STDEV	Ranking	Geomean of ranking values	Final Ranking
0.335	*ACTB*	0.869	*ACTB/HPRT1*	0.516	*HPRT1*	1.445	*TBP*	1.565	*HPRT1*
0.518	*TBP*	0.902	*TBP*	0.816	*ACTB*	1.456	*HPRT1*	1.565	*ACTB*
0.557	*HPRT1*	1.086	*GAPDH*	0.902	*TBP*	1.447	*ACTB*	2.060	*TBP*
1.318	*GAPDH*	1.343	*TUBB3*	0.946	*GAPDH*	1.801	*GAPDH*	4.000	*GAPDH*
1.602	*TUBB3*	1.816	*PPID*	1.473	*TUBB3*	1.996	*TUBB3*	5.000	*TUBB3*
2.584	*PPID*			1.561	*PPID*	2.764	*PPID*	6.000	*PPID*
0.252	*HPRT1*	0.504	*HPRT1/ACTB*	0.419	*GAPDH*	0.842	*HPRT1*	1.189	*HPRT1*
0.317	*ACTB*	0.641	*PPID*	0.534	*HPRT1*	0.915	*ACTB*	2.115	*ACTB*
0.366	*PPID*	0.759	*GAPDH*	0.742	*TBP*	0.921	*PPID*	3.162	*GAPDH*
0.820	*TBP*	0.797	*TBP*	0.840	*PPID*	1.076	*TBP*	3.224	*PPID*
0.865	*GAPDH*	1.083	*TUBB3*	0.854	*ACTB*	1.089	*GAPDH*	3.936	*TBP*
1.580	*TUBB3*			1.581	*TUBB3*	1.655	*TUBB3*	6.000	*TUBB3*
0.135	*GAPDH*	0.438	*GAPDH/TBP*	0.845	*TBP*	0.781	*TBP*	1.189	*TBP*
0.219	*TBP*	0.510	*PPID*	0.847	*PPID*	0.800	*GAPDH*	1.682	*GAPDH*
0.545	*PPID*	0.698	*ACTB*	0.857	*HPRT1*	0.905	*PPID*	2.711	*PPID*
0.715	*ACTB*	0.814	*HPRT1*	0.894	*GAPDH*	1.036	*ACTB*	4.229	*ACTB*
1.015	*HPRT1*	1.025	*TUBB3*	1.021	*ACTB*	1.177	*HPRT1*	4.401	*HPRT1*
1.342	*TUBB3*			1.497	*TUBB3*	1.447	*TUBB3*	6.000	*TUBB3*
0.433	*HPRT1*	0.582	*HPRT1/PPID*	0.682	*ACTB*	0.897	*HPRT1*	1.189	*HPRT1*
0.507	*PPID*	0.690	*GAPDH*	1.085	*HPRT1*	0.910	*PPID*	2.115	*PPID*
0.610	*TBP*	0.776	*TUBB3*	1.213	*TBP*	1.013	*TBP*	3.409	*TBP*
0.667	*GAPDH*	0.850	*TBP*	1.242	*GAPDH*	1.015	*GAPDH*	3.722	*GAPDH*
0.786	*TUBB3*	1.069	*ACTB*	1.250	*PPID*	1.071	*TUBB3*	3.834	*ACTB*
1.386	*ACTB*			1.531	*TUBB3*	1.508	*ACTB*	4.949	*TUBB3*

## Discussion

For reliable and reproducible qPCR analyses, selection of the correct reference gene(s) for a given experimental setup is of paramount importance. EH occurs under both physiologic and pathological conditions. EH plays an essential role during normal fetal development, is a normal response to infection and inflammation, can accompany malignant conditions such as myelofibrosis, and can also occur during chronic inflammation [17]. No previous studies have identified potential reference genes in hematopoietic organs at different stage. In this study, we have described a step-by-step procedure for selection of reference genes to study EH. Genes were selected based on stability analysis in multiple tissues and over multiple developmental time points. We found that the combination of *HPRT1* and *TBP* were the best pair of reference genes for normalization of gene expression in EH studies involving only embryonic or both embryonic and post-natal time points. For studies involving only post-natal time points, the combination of *HPRT1* and *PPID* appear to be the optimal combination.

RNA quality can vary depending on the complexity of the sample, making the choice of RNA extraction methodology a critical component of experimental design. To establish an optimal protocol for the diverse collection of EH organs, we initially compared TRIzol (Invitrogen) (data not shown) and a column-based RNeasy micro kit (Qiagen). TRIzol is a monophasic solution of phenol and guanidinium isothiocyanate that simultaneously solubilizes biological material and denatures protein. The subsequent addition of chloroform causes phase separation where protein RNA is extracted. Other have reported that RNA isolated with TRIzol is often contaminated with genomic DNA, significantly impairing interpretation of RT-PCR results [31]. We also noted poor RNA quality (RIN as low as 1) when using TRIzol (data not shown). The RNeasy micro kit (Qiagen) technology uses selective binding properties of a silica-based membrane to maximize quality of isolated RNA. Using this approach, we noted maximal, or near-maximal RIN with a clean electropherogram for all four organs (Fig 1A and 1B). Other authors have reported difficulties in isolating RNA from certain tissues, including spleen, because of high levels of endogenous nucleases [32]. To overcome this limitation, we utilized BME to prevent RNA degradation in the lysis buffer in all samples. With these approaches, we were able to obtain highly pure RNA (A_260_/A_280_ 1.85–2.25) for further analysis. Finally, because of our interest in embryonic as well as post-natal time points, we were faced with the challenge of small sample amounts. To overcome this limitation, we utilized pre-amplification of cDNA with target specific primer pairs.

Because the embryonic period spans major organogenesis and maturation of tissues and organs during rapid growth of the body, embryonic samples represent potential sources of high variation. Mamo et al [33] showed moderate changes for the genes *ACTB*, *GAPDH*, *HPRT1* and others in an *in vitro* and *in vivo* experiment at different stages using whole embryos. The cells comprising the embryo have a vastly heterogeneous nature, which leads to greater variation in the endogenous biological processes, and greater variation in the sensitivity of cells to their environment [34]. Because these factors can introduce intra- and inter assay variations, we elected to isolate the specific EH organs of interest (heart, liver, spleen, thymus) and analyze gene expression in these organs independently instead of analyzing whole embryo homogenates, as has been previously described [35]. Accordingly, when analyzing variation in Cp, we noted little variability in post-natal samples as compared to embryonic samples (Fig 2). Further, individual genes showed varying degrees of variability, with *TUBB3* highest, and *ACTB* and *GAPDH* demonstrating the least variability.

The analysis of reference genes for studying EH was aided by the use of free and commercially available software packages (NormFinder, geNorm, BestKeeper, DeltaCT, RefFinder) designed for reference gene validation. These software packages differed slightly in the selection of best reference gene or combination of reference genes, emphasizing the importance of understanding the software package’s approach and limitations. RefFinder is a package that integrates the other algorithms and provides a comprenhensive analysis by assigning a weight to each individual gene and calculating the geometric mean of these weights in order to generate a final overall ranking. RefFinder identified *TBP* as the most stable reference gene for embryonic tissue, which echoed the results from NormFinder and the Delta CT. For post-natal and pooled samples (embryonic and post-natal altogether) the most stable reference gene identified by RefFinder was *HPRT1*. Similar results were obtained with geNorm, BestKeeper, and Delta CT method for post-natal, and NormFinder and BestKeeper for pooled samples. Based on these analyses, we propose that the maximal flexibility for studying EH in all of these tissues/times is afforded by using a set of three reference genes. We have identified the best trio of reference genes to allow flexibility of analyses between embryonic and post-natal tissues. *HPRT1*/*TBP*/*GAPDH* were the best genes in combination for embryonic samples, *HPRT1*/*PPID*/*TBP* were the best combination for post-natal samples and *HPRT1*/*TBP*/*GAPDH* were the best trio for all pooled samples.

A limitation of the current study is the use of whole organs rather than specific cells to identify appropriate reference genes through the embryonic and post-natal periods. Indeed, there is a growing literature on the importance of bi-directional cross-talk between hematopoietic stem cells and their surrounding niche cells in both normal myeloid and lymphoid development, as well as in the development of malignancy (36–38). An extension of the current approach would be the use of cell sorting, through either magnetic bead or flow cytometry-based immuno-selection, to separate hematopoietic cells from the surrounding mesenchymal environment. These separate compartments could then be subjected to similar analyses to identify reference genes specific for those cell types of further interest. However, the reference genes we have identified here should prove useful in future studies directed at specific cell-niche interactions in each of these disease processes.

## Conclusions

This is the first study to detail the comprehensive selection of reference genes for normalization of expression in murine extramedullary hematopoietic tissues over multiple developmental stages. We conclude that there are optimal reference genes for individual tissues and developmental stages, but selection of a combination of reference genes affords flexibility in experimental design and analysis. Our study provides the basis for the selection of reference genes for future gene expression studies related to extramedullary hematopoiesis.

## Supporting information

S1 Fig Expression profiles of individual reference genes over different developmental stages. Expression profiles of the individual references genes over the range of developmental stages (E14.5, E16.5, E18.5, P0, P21) in pooled samples (all organs). Mean +/- SEM for 12 samples at each developmental stage is shown.
(TIF)

## References

[1] Yamamoto K, Miwa Y, Abe-Suzuki S, Abe S, Kirimura S, Onishi I, et al Extramedullary hematopoiesis: Elucidating the function of the hematopoietic stem cell niche (Review). Mol Med Rep. 2016;13(1):587–91. doi: 10.3892/mmr.2015.4621 . 26648325 10.3892/mmr.2015.4621

[2] Godin I, Dieterlen-Lievre F, Cumano A. Emergence of multipotent hemopoietic cells in the yolk sac and paraaortic splanchnopleura in mouse embryos, beginning at 8.5 days postcoitus. Proceedings of the National Academy of Sciences of the United States of America. 1995;92(3):773–7. ; 7846049 10.1073/pnas.92.3.773 PMC42702

[3] Medvinsky AL, Samoylina NL, Muller AM, Dzierzak EA. An early pre-liver intraembryonic source of CFU-S in the developing mouse. Nature. 1993;364(6432):64–7. doi: 10.1038/364064a0 . 8316298 10.1038/364064a0

[4] Huyhn A, Dommergues M, Izac B, Croisille L, Katz A, Vainchenker W, et al Characterization of hematopoietic progenitors from human yolk sacs and embryos. Blood. 1995;86(12):4474–85. . 8541536

[5] Tavian M, Coulombel L, Luton D, Clemente HS, Dieterlen-Lievre F, Peault B. Aorta-associated CD34+ hematopoietic cells in the early human embryo. Blood. 1996;87(1):67–72. . 8547678

[6] Migliaccio G, Migliaccio AR, Petti S, Mavilio F, Russo G, Lazzaro D, et al Human embryonic hemopoiesis. Kinetics of progenitors and precursors underlying the yolk sac----liver transition. The Journal of clinical investigation. 1986;78(1):51–60. doi: 10.1172/JCI112572 ; 3722384 10.1172/JCI112572 PMC329530

[7] Golfier F, Barcena A, Cruz J, Harrison M, Muench M. Mid-trimester fetal livers are a rich source of CD34+/++ cells for transplantation. Bone marrow transplantation. 1999;24(5):451–61. doi: 10.1038/sj.bmt.1701940 . 10482927 10.1038/sj.bmt.1701940

[8] Golfier F, Barcena A, Harrison MR, Muench MO. Fetal bone marrow as a source of stem cells for in utero or postnatal transplantation. British journal of haematology. 2000;109(1):173–81. . 10848797 10.1046/j.1365-2141.2000.02009.x

[9] Lobach DF, Haynes BF. Ontogeny of the human thymus during fetal development. Journal of clinical immunology. 1987;7(2):81–97. . 2437143 10.1007/BF00916002

[10] Punnonen J, de Vries JE. Characterization of a novel CD2+ human thymic B cell subset. Journal of immunology. 1993;151(1):100–10. . 7686927

[11] Sanchez MJ, Muench MO, Roncarolo MG, Lanier LL, Phillips JH. Identification of a common T/natural killer cell progenitor in human fetal thymus. The Journal of experimental medicine. 1994;180(2):569–76. ; 7519241 10.1084/jem.180.2.569 PMC2191594

[12] Barcena A, Muench MO, Galy AH, Cupp J, Roncarolo MG, Phillips JH, et al Phenotypic and functional analysis of T-cell precursors in the human fetal liver and thymus: CD7 expression in the early stages of T- and myeloid-cell development. Blood. 1993;82(11):3401–14. . 7694684

[13] Bachvarova R, De Leon V, Johnson A, Kaplan G, Paynton BV. Changes in total RNA, polyadenylated RNA, and actin mRNA during meiotic maturation of mouse oocytes. Developmental biology. 1985;108(2):325–31. . 2416609 10.1016/0012-1606(85)90036-3

[14] Bachvarova R, De Leon V. Polyadenylated RNA of mouse ova and loss of maternal RNA in early development. Developmental biology. 1980;74(1):1–8. . 7350004 10.1016/0012-1606(80)90048-2

[15] Piko L, Clegg KB. Quantitative changes in total RNA, total poly(A), and ribosomes in early mouse embryos. Developmental biology. 1982;89(2):362–78. . 6173273 10.1016/0012-1606(82)90325-6

[16] Sohawon D, Lau KK, Lau T, Bowden DK. Extra-medullary haematopoiesis: a pictorial review of its typical and atypical locations. J Med Imaging Radiat Oncol. 2012;56(5):538–44. doi: 10.1111/j.1754-9485.2012.02397.x . 23043573 10.1111/j.1754-9485.2012.02397.x

[17] Kim CH. Homeostatic and pathogenic extramedullary hematopoiesis. J Blood Med. 2010;1:13–9. doi: 10.2147/JBM.S7224 ; 22282679 10.2147/JBM.S7224 PMC3262334

[18] Glatman Zaretsky A, Silver JS, Siwicki M, Durham A, Ware CF, Hunter CA. Infection with Toxoplasma gondii alters lymphotoxin expression associated with changes in splenic architecture. Infect Immun. 2012;80(10):3602–10. doi: 10.1128/IAI.00333-12 ; 22851754 10.1128/IAI.00333-12 PMC3457551

[19] MacNamara KC, Jones M, Martin O, Winslow GM. Transient activation of hematopoietic stem and progenitor cells by IFNgamma during acute bacterial infection. PLoS One. 2011;6(12):e28669 doi: 10.1371/journal.pone.0028669 ; 22194881 10.1371/journal.pone.0028669 PMC3237486

[20] Rossi MI, Dutra HS, El-Cheikh MC, Bonomo A, Borojevic R. Extramedullar B lymphopoiesis in liver schistosomal granulomas: presence of the early stages and inhibition of the full B cell differentiation. Int Immunol. 1999;11(4):509–18. . 10323203 10.1093/intimm/11.4.509

[21] Jordan S, Ruzsics Z, Mitrovic M, Baranek T, Arapovic J, Krmpotic A, et al Natural killer cells are required for extramedullary hematopoiesis following murine cytomegalovirus infection. Cell Host Microbe. 2013;13(5):535–45. doi: 10.1016/j.chom.2013.04.007 . 23684305 10.1016/j.chom.2013.04.007

[22] Radonic A, Thulke S, Mackay IM, Landt O, Siegert W, Nitsche A. Guideline to reference gene selection for quantitative real-time PCR. Biochem Biophys Res Commun. 2004;313(4):856–62. Epub 2004/01/07. . 14706621 10.1016/j.bbrc.2003.11.177

[23] Dheda K, Huggett JF, Chang JS, Kim LU, Bustin SA, Johnson MA, et al The implications of using an inappropriate reference gene for real-time reverse transcription PCR data normalization. Anal Biochem. 2005;344(1):141–3. Epub 2005/08/02. doi: 10.1016/j.ab.2005.05.022 . 16054107 10.1016/j.ab.2005.05.022

[24] Kubista M, Andrade JM, Bengtsson M, Forootan A, Jonak J, Lind K, et al The real-time polymerase chain reaction. Molecular aspects of medicine. 2006;27(2–3):95–125. doi: 10.1016/j.mam.2005.12.007 . 16460794 10.1016/j.mam.2005.12.007

[25] Vandesompele J, De Preter K, Pattyn F, Poppe B, Van Roy N, De Paepe A, et al Accurate normalization of real-time quantitative RT-PCR data by geometric averaging of multiple internal control genes. Genome Biol. 2002;3(7):RESEARCH0034 ; 12184808 10.1186/gb-2002-3-7-research0034 PMC126239

[26] Andersen CL, Jensen JL, Orntoft TF. Normalization of real-time quantitative reverse transcription-PCR data: a model-based variance estimation approach to identify genes suited for normalization, applied to bladder and colon cancer data sets. Cancer Res. 2004;64(15):5245–50. Epub 2004/08/04. doi: 10.1158/0008-5472.CAN-04-0496 . 15289330 10.1158/0008-5472.CAN-04-0496

[27] Pfaffl MW, Tichopad A, Prgomet C, Neuvians TP. Determination of stable housekeeping genes, differentially regulated target genes and sample integrity: BestKeeper—Excel-based tool using pair-wise correlations. Biotechnol Lett. 2004;26(6):509–15. Epub 2004/05/07. . 15127793 10.1023/b:bile.0000019559.84305.47

[28] Silver N, Best S, Jiang J, Thein SL. Selection of housekeeping genes for gene expression studies in human reticulocytes using real-time PCR. BMC molecular biology. 2006;7:33 doi: 10.1186/1471-2199-7-33 ; 17026756 10.1186/1471-2199-7-33 PMC1609175

[29] Xie F, Xiao P, Chen D, Xu L, Zhang B. miRDeepFinder: a miRNA analysis tool for deep sequencing of plant small RNAs. Plant molecular biology. 2012 doi: 10.1007/s11103-012-9885-2 . 22290409 10.1007/s11103-012-9885-2

[30] Schroeder A, Mueller O, Stocker S, Salowsky R, Leiber M, Gassmann M, et al The RIN: an RNA integrity number for assigning integrity values to RNA measurements. BMC molecular biology. 2006;7:3 doi: 10.1186/1471-2199-7-3 ; 16448564 10.1186/1471-2199-7-3 PMC1413964

[31] Mannhalter C, Koizar D, Mitterbauer G. Evaluation of RNA isolation methods and reference genes for RT-PCR analyses of rare target RNA. Clin Chem Lab Med. 2000;38(2):171–7. doi: 10.1515/CCLM.2000.026 . 10834406 10.1515/CCLM.2000.026

[32] Pinkert CA. Transgenic animal technology: a laboratory handbook: Newnes; 2014.

[33] Mamo S, Gal AB, Bodo S, Dinnyes A. Quantitative evaluation and selection of reference genes in mouse oocytes and embryos cultured in vivo and in vitro. BMC Dev Biol. 2007;7:14 doi: 10.1186/1471-213X-7-14 ; 17341302 10.1186/1471-213X-7-14 PMC1832186

[34] Zhang QJ, Chadderton A, Clark RL, Augustine-Rauch KA. Selection of normalizer genes in conducting relative gene expression analysis of embryos. Birth defects research Part A, Clinical and molecular teratology. 2003;67(8):533–44. doi: 10.1002/bdra.10075 . 14632301 10.1002/bdra.10075

[35] Brawand D, Soumillon M, Necsulea A, Julien P, Csardi G, Harrigan P, et al The evolution of gene expression levels in mammalian organs. Nature. 2011;478(7369):343–8. doi: 10.1038/nature10532 . 22012392 10.1038/nature10532

