# Supplementary figures and images for: Comprehensive selection of reference genes for quantitative RT-PCR analysis of murine extramedullary hematopoiesis during development
Source: PLoS One. 2017 Jul 21;12(7):e0181881. doi: 10.1371/journal.pone.0181881 (PMC5521956; doi:10.1371/journal.pone.0181881)

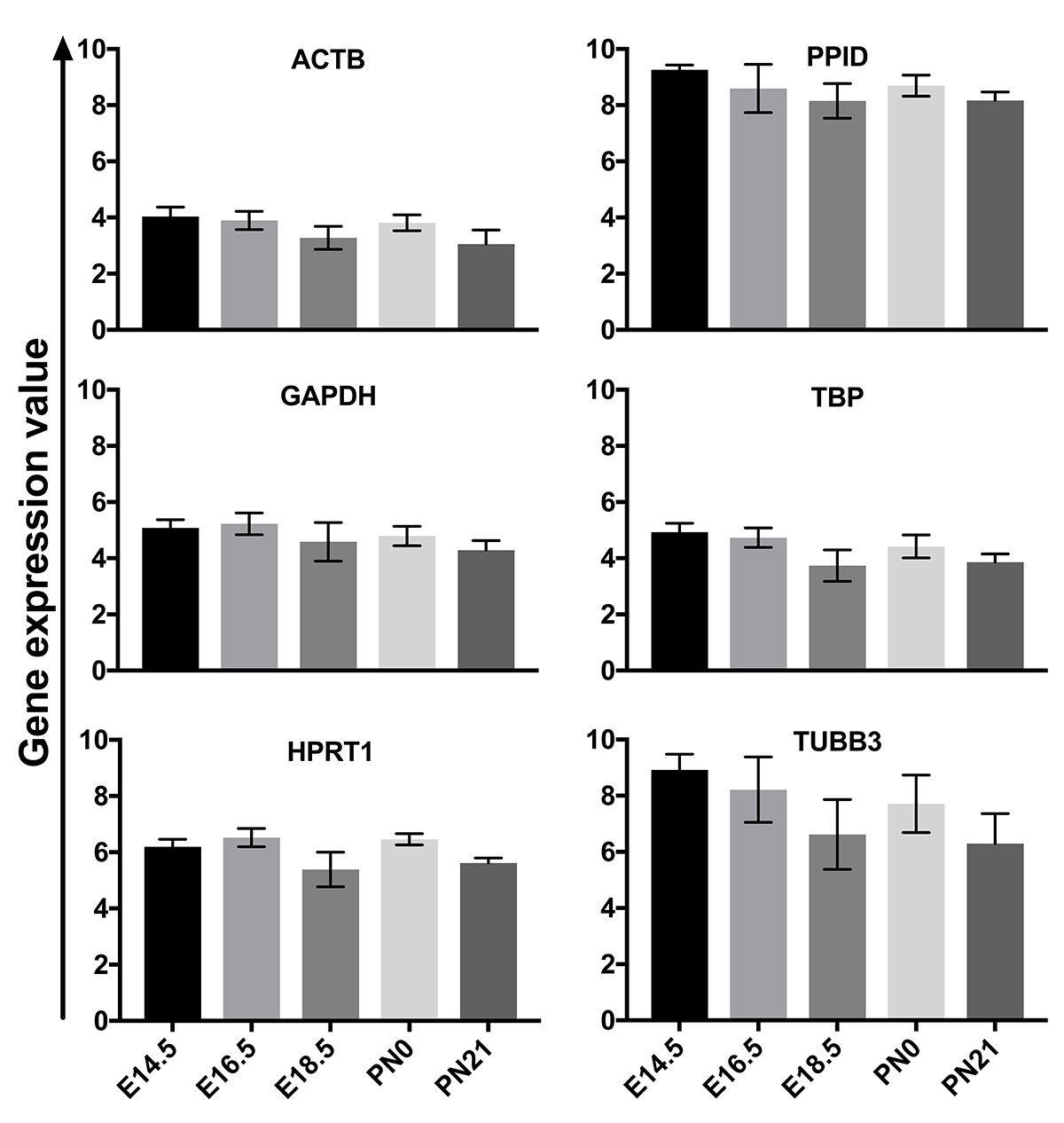

Supplement: S1 Fig — Expression profiles of the individual references genes over the range of developmental stages (E14.5, E16.5, E18.5, P0, P21) in pooled samples (all organs). Mean +/- SEM for 12 samples at each developmental stage is shown. (TIF) [file pone.0181881.s001.tif]
